# La situación actual de la Medicina de Laboratorio en España

**DOI:** 10.1515/almed-2022-0005

**Published:** 2022-03-03

**Authors:** Imma Caballé

**Affiliations:** Sociedad Española de Medicina de Laboratorio, Barcelona, Spain; Catlab, Viladecavalls, Spain

**Keywords:** laboratorio clínico, Libro Blanco, profesionales

Durante la pandemia de COVID-19, el papel de la Medicina de Laboratorio ha sido y sigue siendo crucial. A efectos de la población general, se ha reflejado su contribución decisiva a la salud poblacional, y a su vez también se han evidenciado las limitaciones y nuevas necesidades emergentes.

Sorprendentemente, el conocimiento objetivo de la situación de la Medicina de Laboratorio en España dista mucho de ser el deseable. La información estadística es escasa y parcial. Actualmente no hay un catálogo único de prestaciones que incorpore la misma nomenclatura y medición de la actividad, por este motivo resulta difícil saber el volumen de actividad, el coste, la eficiencia, organización, calidad y la dotación de profesionales de los laboratorios clínicos.

Es por ello, que desde la Sociedad Española de Medicina de Laboratorio se emprendió la iniciativa de elaborar un Libro Blanco [1] que reflejara la situación y permitiera de esta forma dar respuesta a las limitaciones existentes. Un Libro Blanco pretende mostrar una descripción en un momento del tiempo, y se tomó el año 2019 como referencia, antes del inicio de la pandemia.

El esfuerzo realizado por todos los participantes en esta iniciativa ha sido notable. La cumplimentación del cuestionario obligaba a revisar y actualizar los datos de cada laboratorio. En un inicio se tuvo que determinar el listado de laboratorios disponible, ya que a fecha de hoy no hay una base de datos fiable y actualizada sobre el número de los laboratorios en funcionamiento. Por este motivo se trataba de garantizar la máxima representatividad, por volumen de actividad y geografía. El Libro Blanco incluye los datos relativos a 174 laboratorios clínicos, los cuales han informado de 55,9 millones de peticiones y 800 millones de determinaciones. Se estima que ofrece una representatividad del 90% de las determinaciones realizadas en los Laboratorios Clínicos. Ahora bien, en próximas ediciones todavía habrá que mejorar en la calidad de la información.

Una cuestión altamente relevante es la relativa a la situación de los profesionales. La cantidad y tipo de profesionales en la Medicina de Laboratorio era una cifra desconocida hasta el momento ya que los datos que poseía el Ministerio solo contemplaban a los médicos. El Libro Blanco refleja el estado de situación y ofrece mensajes claros para la toma de decisiones por parte del gobierno. Los laboratorios clínicos en España tienen una necesidad de plazas para renovar la plantilla que implicará en torno al 20–25% de los titulados en los próximos 5 años (el 23% de los titulados supera los 60 años). Con una mirada más amplia, en los próximos 15 años será necesario renovar más de la mitad de la plantilla de los laboratorios clínicos, por lo que, si las planificaciones de oferta de plazas de formación especializada no se adecúan en tiempo a la demanda prevista, en muy poco tiempo se empezará a notar la extrema escasez de profesionales en el sector.

Al mismo tiempo el Libro Blanco enfatiza en la necesidad de unificación de las especialidades de Análisis Clínicos y Bioquímica Clínica en una especialidad única de que agrupe la formación especializada y aumente la oferta de puestos de trabajo. En Europa, la situación es diversa y así lo resume un artículo reciente [2].

Una cuestión clave es asimismo poder demostrar la calidad de nuestros resultados que ayudan a los médicos a tomar buenas decisiones clínicas y un referente para ello es la Certificación ISO. Según la encuesta realizada, solo el 49% de ellos cumplen la Certificación ISO 9001 y solo 85 laboratorios españoles cumplen la Acreditación ISO 15189. Por este motivo la SEQC aboga por la obligatoriedad de la Acreditación ISO 15189, la más importante del sector de los laboratorios clínicos que establece la fiabilidad de nuestros resultados y que es de obligado cumplimiento en muchos países europeos.

La innovación tecnológica representa también uno de los factores determinantes de la evolución futura del Laboratorio Clínico. El nivel de automatización creciente que se ha conseguido en los laboratorios representa un hito destacable, permitiendo una mayor eficiencia. De hecho, los centros encuestados han realizado una intensa labor de incorporación de tecnologías digitales y de automatización en sus modelos operativos: el 90% de los centros alcanzan la automatización de más del 75% de sus pruebas y el 82% de los centros cuentan con la petición electrónica de más del 75% de sus pruebas.

La Medicina de Laboratorio ejercerá un papel protagonista en lo que se ha denominado la Bio-revolución [3]. La biología molecular y la genómica está ofreciendo nuevas oportunidades de secuenciación que permiten realizar pruebas que antes eran realizadas en el ámbito de la investigación. La aplicación de Big data y de la Inteligencia Artificial en el Laboratorio Clínico será de utilidad en ámbitos como la fijación de los valores de referencia, garantía de calidad, apoyo a decisiones clínicas y monitorización de salud poblacional. Estas innovaciones impulsan al mismo tiempo la necesidad de redefinir el enfoque de la profesión. Este debate a nivel europeo ya se ha iniciado [4].

Por último, el compromiso medioambiental es cada vez más relevante en los laboratorios. Según los datos recogidos en el cuestionario, a nivel nacional, aproximadamente un 90% de los laboratorios posen un programa de gestión medioambiental y un 98% poseen un protocolo de gestión de residuos. Pero una asignatura pendiente es el tratamiento de los residuos líquidos que genera el laboratorio.

Este Libro Blanco ha sido el fruto de una colaboración de los miembros de la Sociedad Española de Medicina de Laboratorio y deseamos que su difusión contribuya a la mejora y a la capacidad de afrontar los retos inmediatos y del próximo futuro.
